# *SUnSeT*: *s*pectral *un*mixing of hyperspectral images for phenotyping soybean *se*ed *t*raits

**DOI:** 10.1007/s00299-024-03249-0

**Published:** 2024-06-09

**Authors:** Seok Won Jeong, Jae Il Lyu, HwangWeon Jeong, Jeongho Baek, Jung-Kyung Moon, Chaewon Lee, Myoung-Goo Choi, Kyoung-Hwan Kim, Youn-Il Park

**Affiliations:** 1 Biological Sciences, Chungnam National University, 99 Daehagro, Youseong, Daejon 34134 Korea; 2 Gene Engineering Division, National Institute of Agricultural Sciences, 370 Nongsaengmyeongro, Jeonju, Jeollabuk-do 54874 Korea; 3 Crop Foundation Research Division, National Institute of Crop Sciences, 181 Hyeoksinro, Wanju, Jeollabuk-do 55365 Korea; 4 Crop Cultivation and Environment Research Division, National Institute of Crop Sciences, 54 Seohoro, Suwon, Kyounggi-do 16613 Korea; 5 Wheat Research Team, National Institute of Crop Sciences, RDA, 181 Hyeoksinro, Wanju, Jeollabuk-do 55365 Korea

**Keywords:** Endmembers, Genome-wide association study, Hyperspectral images, Korean soybean core collection, Linear discriminant analysis, Seed phenotyping, Spectral unmixing

## Abstract

****Key message**:**

**Hyperspectral features enable accurate classification of soybean seeds using linear discriminant analysis and GWAS for novel seed trait genes**.

**Abstract:**

Evaluating crop seed traits such as size, shape, and color is crucial for assessing seed quality and improving agricultural productivity. The introduction of the **SUnSet** toolbox, which employs hyperspectral sensor-derived image analysis, addresses this necessity. In a validation test involving 420 seed accessions from the Korean Soybean Core Collections, the pixel purity index algorithm identified seed- specific hyperspectral endmembers to facilitate segmentation. Various metrics extracted from ventral and lateral side images facilitated the categorization of seeds into three size groups and four shape groups. Additionally, quantitative RGB triplets representing seven seed coat colors, averaged reflectance spectra, and pigment indices were acquired. Machine learning models, trained on a dataset comprising 420 accession seeds and 199 predictors encompassing seed size, shape, and reflectance spectra, achieved accuracy rates of 95.8% for linear discriminant analysis model. Furthermore, a genome-wide association study utilizing hyperspectral features uncovered associations between seed traits and genes governing seed pigmentation and shapes. This comprehensive approach underscores the effectiveness of **SUnSet** in advancing precision agriculture through meticulous seed trait analysis.

**Supplementary Information:**

The online version contains supplementary material available at 10.1007/s00299-024-03249-0.

## Introduction

Seeds are an important source of food for human beings and confer genetic backgrounds for crop yield (TeKrony and Egli 1991). Various phenotyping tools have been used to evaluate seed quality in a quantitative manner. Among these tools, manual inspection has been de facto standard. However, this is notorious for being error-prone, laborious, and time-consuming, especially for massive number of seeds. To amend this, several high-throughput phenotyping methods have been recently developed to generate more precise and reproducible quantitative measurements of seeds.

One prominent phenotyping method is using image sensors and computer-based analysis tools, which has largely increased accuracy particularly for plants and specifically for seeds. Utilizing 2D and 3D images primarily captured by red-green-blue (RGB) cameras, various computer vision algorithms have been employed to extract diverse agricultural traits from seeds. Noteworthy software applications such as **SmartGrain** (Tanabata et al. 2012) and **SeedExtractor** (Zhu et al. 2021) have been developed for seed phenotyping, focusing on aspects such as seed shape and color. To complement RGB-based images, which capture only surface properties, X-ray-based imaging methods (Gagliardi and Marcos-Filho 2011; Gomes-Junior et al. 2012) have been introduced to examine seed structure and germination capacity. This addresses the limitation of RGB cameras, which provide low spectral resolution by acquiring only three wavelengths (R, G, B), neglecting other wavelengths rich in chemical composition (Yang et al. 2015). Despite the benefits, X-ray imaging is often time-consuming, rendering it unsuitable for high-throughput phenotyping (Dumont et al. 2015).

Hyperspectral imaging sensors provide both spatial and spectral information and enough throughput for real-world scenarios. With a hyperspectral sensor, reflectance spectra across a broad range of wavelengths, including ultraviolet (UV), visible (VIS), and near-infrared (NIR) spectra, can be obtained using various platforms (Bock et al. 2010). Due to its ease of operation and scalability, hyperspectral imaging has become a widely accepted tool, supplanting RGB cameras. Some examples are assessing the viability of wheat seeds (Zhang et al. 2018), classifying rice seed cultivars (Qiu et al. 2018), and determining the starch content of corn seeds (Liu et al. 2020). A recent development in this domain is the **HyperSeed** platform, designed to provide hyperspectral information for seeds (Gao et al. 2021). This software offers a comprehensive solution for high-throughput seed phenotyping with HSI techniques. It includes a lab-based imaging system coupled with an application featuring a graphical user interface (GUI) implemented in MATLAB, an open-source platform available to researchers with an institutional license.

The data representing phenotypic characteristics, extracted from hyperspectral images (HSI), has been integrated with genotype data typically comprising genome-wide single nucleotide polymorphisms (SNPs) identified through various methods such as resequencing, genotyping-by-sequencing, or array-based genotyping. This integration facilitates several genetic analyses including genomic prediction (Krause et al. 2019; Sandhu et al. 2021), as hyperspectral patterns and spectra indices are genetically linked with target phenotypes. Moreover, it enables genetic inference studies such as genome-wide association (GWA), heritability, and genetic correlation analyses (Feng et al. 2017; Sun et al. 2019; Barnaby et al. 2020; Wu et al. 2021; Massahiro Yassue et al. 2023) to explore allelic variation.

Among the wide variety of crop seeds, soybean (*Glycine max* (L.) Merr.) stands as a crucial food and feed crop, being the world’s most cultivated annual herbaceous legume. Recognizing its significance, efforts have been made to construct soybean core collections (Oliveira et al. 2010; Jeong et al. 2019; Kim et al. 2022; Jo et al. 2023; Nair et al. 2023). Core collection is a subset that represents the genetic and phenotypic diversities of the entire collection (Frankel and Frankel 1984). Utilizing a core collection is advantageous for conserving and harnessing the genetic variability of soybeans in research and breeding programs. Among several soybean core collections, the Korean soybean core collection (KSCC) was meticulously assembled, leveraging genotypic and phenotypic data, as well as population structure analysis. The KSCC comprises 430 accessions carefully selected from a pool of 2,872 collections, based on Affymetrix Axiom®180k SoyaSNP array data (Jeong et al. 2019).

Despite the paramount importance of seed traits, the initial focus was primarily on investigating morphological characteristics, seed weights (Baek et al. 2020; Nair et al. 2023), shape and colors (Baek et al. 2020; Kim et al. 2022; Nair et al. 2023). Unfortunately, in the previous reports, seed shape and colors were not characterized in a whole core-collection wide, which hampers prediction or classification of core collection using machine learning algorithms. In previous approaches to soybean seed phenotyping, seed morphological traits were extracted from 2D RGB images using the software ImageJ. However, this method proved to be laborious, time-consuming, and error-prone, resulting in lower reproducibility. Additionally, seed coat colors for all KSCC accessions were not classified, even through manual inspection. To overcome these challenges, there is a need for a precise and reproducible phenotyping method based on a command line interface (CLI) for classification.

Machine learning, a subdomain of Artificial Intelligence (AI), encompasses algorithms capable of deriving valuable insights from data and utilizing this information in self-learning processes for effective classification or prediction. Advancements in hardware and software components within machine vision systems have propelled the popularity of machine learning algorithms with fair reliability and accuracy. These algorithms process data swiftly and deliver reliable decisions in minimal time. Various techniques like Artificial Neural Networks (ANN), Fuzzy logic, decision trees, Naïve Bayes, k-means clustering, support vector machines (SVM), random forest (RF), k-Nearest Neighbor (k-NN), among others, have found extensive use in agricultural-related fields (Rehman et al. 2019; Saha and Manickavasagan 2021).

Linear Discriminant Analysis (LDA) is a linear transformation method that reduces the dimensions within a dataset to identify a feature subspace optimizing class separability. While LDA assumes the normal distribution of data and statistical independence of features, it can be applied to datasets even when these assumptions are not entirely met (Raschka and Mirjalili 2017). Primarily used for feature extraction, LDA enhances computational efficiency and reduces overfitting. Its robustness has led to widespread applications in agricultural product classification based on hyperspectral data (Mahesh et al. 2008; Liu et al. 2010; Delwiche et al. 2011; Qin et al. 2020). Most studies employing LDA for hyperspectral imaging-based classification report an average accuracy exceeding 90%, affirming the classifier’s robustness.

According to UPOV guidelines, soybean seed classification includes three main categories: seed weight, seed shape, and seed testa colors, with 3, 4, and 7 different classes, respectively (UPOV, https://www.upov.int/edocs/mdocs/upov/en/twa_46/tg_80_7_proj_3.pdf). This theoretically yields 84 input features. However, in an initial investigation, machine learning of KSCC seeds classification using these features led to underfitting with a lower accuracy of 25%. Therefore, additional features or predictors are required for machine learning.

In this study, we aimed to address this challenge by conducting seed classification based on features extracted from hyperspectral analysis using 420 KSCC accessions as the test case. We overcame the limitations of manual and GUI-dependent methods in quantifying seeds with morphological and biochemical changes by extracting four endmembers using the pixel purity index (PPI) algorithm. These endmembers helped segment seeds from backgrounds and distinguish the seed testa region from the hilum. RGB triplet codes and grayscale values for seed accessions were then obtained from the segmented seed testa. A total of 199 features were extracted from both morphological and hyperspectral traits for classification. We applied LDA to classify the 420 KSCC accessions, achieving high accuracy compared to other machine learning classifiers. Averaged seed coat reflectance in the range of 450 to 900 nm may be useful for characterizing soybean seed accessions. We have developed a toolbox called **SUnSet** (**S**pectral **Un**mixing of hyperspectral images for phenotyping soybean **Se**ed **T**raits). This toolbox encompasses spectral unmixing, seed segmentation, feature extraction, model production using LDA classifier, and screening genes that determine these seed traits. **SUnSet** serves as a potential tool for comprehending genetic variation and expediting agricultural trait enhancement of soybean seeds by identifying genes associated with controlling seed traits.

## Materials and methods

### Workflow

**Fig. 1:**
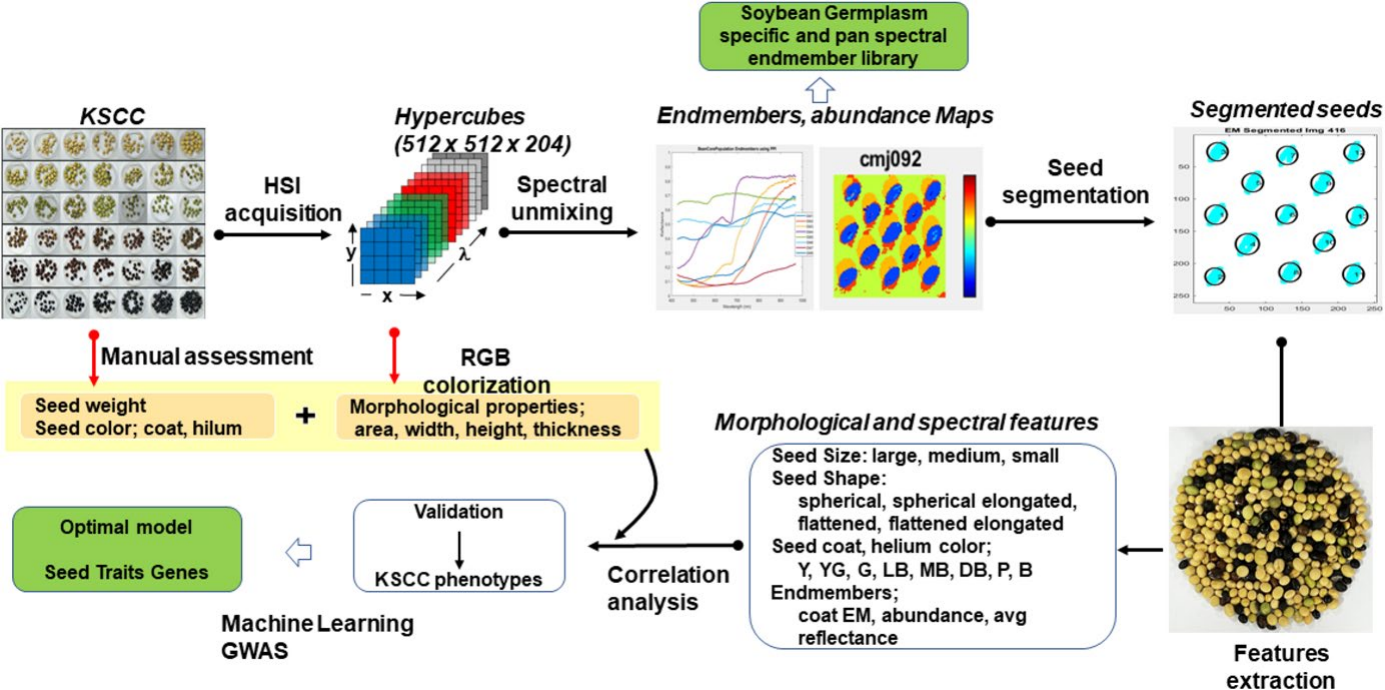
Overview of the key steps in hyperspectral image acquisition, feature extraction, and classification of soybean seed germplasms using the **SUnSet** toolbox integrated with Genome-Wide Association Study (GWAS)

The workflow of the current work is illustrated in Fig. 1. Each KSCC accession with 15 to 50 seeds is placed under the illumination of two halogen lamps (750W, ARRILITE Plus 750), and HSI are generated in the form of a hypercube. Seed-specific endmembers were extracted and then used to segment seeds from the background. Seed-based morphological and hyperspectral features were extracted for further analysis. Seed weight and morphological features such as length, area, and shape of seeds, extracted from reconstituted RGB images from hypercubes using ImageJ software (ImageJ 1.54d, http://imagej.org), were used for validation. Machine learning algorithms were utilized to classify KSCC seeds into their respective accessions. The entire process, from data analysis to machine learning, was conducted using the **SUnSet** toolbox, which is implemented in MATLAB (The MathWorks, Inc., Natick, USA). Additionally, Genome-Wide Association Study (GWAS) was employed to screen candidate genes controlling seed traits.

### Sample preparation and HSI acquisition

Seeds were harvested from 420 KSCC accessions grown at National Institute Agricultural Science (NIAS, Jeonju, Korea) during the 2022 harvest season. For each seed accession, HSI were acquired for the ventral or lateral sides of the seeds. Fifteen or fifty seeds per accession were used to extract morphological and hyperspectral features, respectively. The hyperspectral camera and light source were mounted on a camera tripod stand (Manfrotto 155), with a lamp case attached to the stand (ARRILITE 750 Plus, München, Germany). A high-performance line-scan image spectrograph, SPECIM IQ (Specim Ltd, Oulu, Finland), was fixed on top of the stand, covering the spectral range from 400 to 1,000 nm with a 2.9 nm spectral resolution (Behmann et al. 2018). The fore optic of the camera provided a 31^∘^ x 31^∘^ field of view, and the minimum working distance of the camera lens was 150 mm. Two lighting units with a 750 W tungsten-halogen bulb (Ushio HPL X Plus Long Life Lamp, 750W/230V, color temperature 3,050K) were fixed on top of the frame to illuminate the seed samples, and the light intensity on the top of the seeds was 1,700 $\mu$mol m^-2^s^-1^.

Image acquisitions were performed in a dark room at room temperature. The distance and exposure time were set to 16 cm and 1 ms, respectively, resulting in a spatial resolution of 0.17 mm and 0.20 mm on the targets for ventral and lateral view images, respectively. On average, it took 30 s to capture one image in the form of three-dimensional hypercubes of 512 x 512 pixels x 204 wavelengths. For the measurement of the white reference, a 99% barium sulfate white panel was recorded simultaneously with the seeds.

### Spectral unmixing for identifying KSCC seed specific endmembers

HSI, compatible with the ENVI format and consisting of pairs of raw images (*.raw) and header (*.hdr) files, were radiometrically corrected using the corresponding software Specim IQ Studio (Behmann et al. 2018). After data preprocessing, the remaining procedures were executed using Matlab R2023b, along with the Computer Vision Toolbox, Imaging Process Toolbox, and Parallel Computing Toolbox. First, hypercubes were generated, visualized in RGB images, and then regions of interest (ROIs) containing seeds were cropped out, excluding the whiteboard panel. The number of endmembers present in a hyperspectral data cube was determined by using the noise-whitened Harsanyi-Farrand-Chang (NWHFC) method with a probability false alarm value of $10^{-3}$ (Chang and Du 2004).

Pixel Purity Index (PPI) algorithm that is used to identify the most spectrally pure (extreme) pixels in remote-sensed images was applied to the Minimum Noise Fraction (MNF) Rotation transform output image to select spectral endmembers (Boardman et al. 1995). Spectral variability of endmembers caused by variable illumination and environmental, atmospheric, and temporal conditions (Somers et al. 2011) was computed from the mean-squared error (MSE) values obtained for each endmember spectrum with respect to all the endmembers from initially extracted endmember dataset.

1 $$\begin{aligned} \text {MSE} = \frac{1}{n} \sum _{i=1}^{n} (m_i - \hat{m}_i)^2 \end{aligned}$$

where $m_i$ and $\hat{m}_i$ are reference and test endmember i, and n is the number of wavebands. Endmembers with spectrally similar characteristics were chosen based on the MSE values within the rage 0 < MSE < 5 x 10^-5^. The resulting averaged spectra was then regarded as the set of endmembers for HSI analysis. Subsequently, the proportion of each endmember present in the spectra of each pixel was estimated as abundance maps. For a hyperspectral data cube of spatial dimensions *M*-by-*N* containing *P* endmembers, there exist *P* abundance maps, each of size *M*-by-*N*.

To quantify the accuracy of the reconstruction over the original image, the relative root mean square error (rRMSE) value was determined using the formula:

2 $$\begin{aligned} \text {rRMSE(X)} = \frac{\sqrt{\frac{1}{n} \sum _{i=1}^{n}(x_i - x_i^{\text {ref}})^2}}{\text {max}(\text {X}^{\text {ref}}) - \text {min}(\text {X}^{\text {ref}})} x 100\% \end{aligned}$$

where X is a reconstructed image, X^ref^ is an original reference image, *n* is the number of pixels, max and min is the maximum and minimum value, respectively (Chen et al. 2012).

To assess similarity among endmembers, the spectral angle mapper (SAM) classification algorithm (Kruse et al. 1993), the spectral information divergence (SID) technique (Chang 2000), and the normalized spectral similarity score method (NS3) (Nidamanuri and Zbell 2011) were utilized. Given the test spectra *t* and a reference spectra *r* of length n, SAM, SID and NS3 scores that computes spectral similarity based on the Euclidean and SAM distances between two spectra are calculated as following equations

3 $$\begin{aligned} \text {SAM}= & {} \cos ^{-1} \left( \frac{\sum _{i=1}^{n} t_i r_i}{\sqrt{\sum _{i=1}^{n} t_i^2} \sqrt{\sum _{i=1}^{n} r_i^2}} \right) \end{aligned}$$

4 $$\begin{aligned} \text {SID}= & {} \sum _{i=1}^{n} t_i \log \left( \frac{t_i}{r_i}\right) + \sum _{i=1}^{n} r_i \log \left( \frac{r_i}{t_i}\right) \end{aligned}$$

5 $$\begin{aligned} \text {Euclidean}= & {} \sqrt{\frac{1}{n} \sum _{i=1}^{n} (t_i - r_i)^2} \end{aligned}$$

6 $$\begin{aligned} \text {NS3}= & {} \sqrt{\text {Euclidean}^2 + (1 - \cos (\text {SAM}))^2} \end{aligned}$$

where $t_{i}$ and $r_{i}$ are the $\textit{i}$-th elements of the vectors t and r, respectively.

Vegetation indices for vegetation (NDVI; Schnell 1974), chlorophyll (TCARI; Haboudane et al. 2004) and anthocyanin (ARI2; Gitelson et al. 2001) were obtained by formula as followings.

7 $$\begin{aligned} \text {NDVI}= & {} \frac{R_{800} - R_{680}}{R_{800} + R_{680}} \end{aligned}$$

8 $$\begin{aligned} \text {ARI2}= & {} R_{800} \left( \frac{1}{R_{550}} - \frac{1}{R_{700}} \right) \end{aligned}$$

9 $$\begin{aligned} \text {TCARI}= & {} 3\left[ (R_{700} - R_{670}) - 0.2(R_{700} - R_{550}) \left( \frac{R_{700}}{R_{670}}\right) \right] \end{aligned}$$

where the symbols $R_{800}$, $R_{680}$, $R_{550}$, $R_{700}$, $R_{670}$ represent reflectance at wavelengths 800nm, 680nm, 550nm, 700nm, and 670nm, respectively.

### Seed segmentation

Intensity thresholding techniques, commonly employed in imaging analysis, have been extensively used for background removal in seed segmentation (Baek et al. 2020; Gao et al. 2021). In preliminary study, the hypercube image was initially reduced to a 3D RGB image, segmented using GUI-dependent color thresholders or CLI-dependent single or multiple thresholding values. However, applying these thresholding techniques for KSCC seed segmentation posed challenges due to difficulty in distinguishing background colors overlapping with seed testa or hilum colors, exacerbated by unavoidable shadowing caused during image capture. Additionally, the underutilization of spectral information in the NIR regions, despite using relatively expensive hyperspectral sensors, rendered these techniques less justifiable.

In this study, specific endmembers for seed foregrounds or backgrounds were visually inspected and grouped based on the abundance maps. Pixels associated with foreground endmembers underwent further processing. To isolate overlapped seeds into individual entities, RGB codes were converted into grayscale(GS) using a weighted average formula ($GS = 0.299R + 0.587G + 0.114B$). Subsequently, gradient magnitude in both horizontal and vertical directions was computed using a Sobel kernel (Kanopoulos et al. 1988), and Otsu’s method (Otsu 1979) was applied to convert the grayscale image into a binary image using a global threshold. Morphological operation using opening filter (van den Boomgaard and van Balen 1992) were then conducted to first shrink and then expand foreground objects to approximately their original size using a square structuring element.

Following this, MATLAB’s *regionprops* function was utilized to measure properties such as area, major axis, minor axis, and centroid for each 8-connected component in the binary image. After filtering components out based on their size compared to the median value, a series of masks representing individual seeds were obtained. This process achieved a ratio of 1.0 between the number of imaged seeds and the number of segmented seeds, indicating minimal misinterpretation of background pixels as seeds. Additionally, the potential issue of multiple seeds being treated as a single entity due to overlapping was successfully mitigated. Seed shape indices, including width/height and thickness/width ratios, were calculated from ventral side area, width, and height, along with lateral side thickness measurements.

### RGB triplet and hyperspectral data extraction

Soybean seeds are categorized into eight different classes based on the color of the seed testa, specifically yellow, yellow-green, green, light brwon, intermediate brown, dark brown, and black seeds, following the UPOV guideline. An RGB triplet in the range [0, 1] is a three-element row vector specifying the intensities of the red, green, and blue components of the chosen color. To obtain the RGB triplet for each seed accession, an initial visual inspection of seed colors was conducted by ten individuals. In cases of disagreement on classification, the class with the majority of votes was assigned to the seed coat color.

For the segmentation of the seed coat from the hilum, pixels with the most abundant endmember were selected. These refined seed segmentation take the form of binary masks, consisting of zero and non-zero values. Subsequently, these masks were applied to the x-y plane of the hypercube. Pixels with zero values in the mask were set to zero, while all others remained unchanged in the output image. Additionally, due to the low sensitivity of camera sensors at the spectral extremes, outliers caused by random noise often appear. For improved accuracy, 5% of bands at the beginning and end were removed in this study, resulting in the selection of spectral bands with wavelengths in the range of 426–970 nm. Following the refinement of the hypercube, RGB triplets and averaged reflectance spectra of pixels within each segmented seed were extracted. This process ensures a more accurate representation of soybean seed characteristics, considering both color information and spectral reflectance.

### Data transformation

Reflectance spectra in close-range imaging are often influenced by the illumination and background environment. This influence has been effectively mitigated by employing derivative spectra (Zhang et al. 2006; Prasad and Gnanappazham 2014). First-order derivative spectra (dR)  reveal the peak characteristics of the spectrum, reflecting waveform changes caused by the absorption of light by chlorophyll and other substances in plants (Becker et al. 2005). Additionally, logarithmic transformation (logR) (can enhance spectral differences in the visible region and reduce the impact of multiplicative factors caused by changes in illumination conditions (Pu and Gong 2011). dR and logR  values were obtained by the following formulas.

10 $$\begin{aligned} \text {dR} &= \left[ \frac{r_3 - r_1}{\Delta \lambda }, \frac{r_4 - r_2}{\Delta \lambda }, \ldots , \frac{r_n - r_{n-2}}{\Delta \lambda } \right] \end{aligned}$$

11 $$\begin{aligned} \text {logR}= & {} [\log (r_1), \log (r_2), \ldots , \log (r_n)] \end{aligned}$$

where *ri* denotes reflectance at the *i*-th wavelength, *n* denotes the number of wavebands, and $\Delta \lambda$ denotes the waveband intervals (nm).

### Seed classification

The classification of KSCC accessions based on seed size, shape, and colors was conducted using machine learning models. Seed sizes were categorized into three classes–small, medium, and large–utilizing weight per seed. As weight-dependent seed size classification is not feasible using 2D images, the variables of area, width, height, and thickness were employed instead. Seed shapes were classified into four types–spherical, spherical flattened, elongated, and elongated flattened–based on the ratios of width/height and thickness/width. Seed coat colors for the core collection were manually categorized into seven different colors: yellow, yellow-green, green, light brown, medium brown, dark brown, and black, respectively. Quantitative RGB triplet codes for the KSCC accessions were obtained from seed testa segmentation based on testa-abundant endmembers. Additionally, averaged reflectance values at given wavelengths and spectral indices such as NDVI, ARI2, and TCARI of every seed were included to produce features for machine learning.

The final dataset comprised 420 response classes (KSCC seed accessions) and 199 predictors, including seed size, shape, color code, RGB triplet, averaged entire spectrum, and pigment chlorophyll and anthocyanin indices derived from reflectance spectra. This dataset was divided into two parts: 80% of seeds for training and 20% of seeds for testing, using random selection. To classify KSCC accession seeds, the entire set of methods in the classification learner toolbox, consisting of 31 machine learning algorithms available in MATLAB 2023b software, was utilized. The selected parameters are default parameters of the MATLAB 2023b classification learner tool; therefore, no classifiers were optimized to achieve higher classification accuracy.

Five-fold cross validation with default settings is used with these classifiers to obtain numerical results. Models obtained as such are evaluated using four performance metrics on the test samples: Accuracy, Precision, Recall, and F1-score. Usually higher values represent better performance and formula are given as Eqs. (9)–(12).

12 $$\begin{aligned} \text {Accuracy (A)}= & {} \frac{(\text {TP} + \text {FP)}}{(\text {TP + TN + FP + FN})} \end{aligned}$$

13 $$\begin{aligned} \text {Precision (P)}= & {} \frac{\text {TP}}{\text {(TP + FP)}} \end{aligned}$$

14 $$\begin{aligned} \text {Recall (R)}= & {} \frac{\text {TP}}{\text {(TP + FN)}} \end{aligned}$$

15 $$\begin{aligned} \text {F1-score}= & {} \frac{\text {2PR}}{\text {(P + R)}} \end{aligned}$$

where TP, FP, TN, and FN are true positives, false positives, true negatives and false negatives, respectively. Moreover, the model is also evaluated using seed group prediction accuracy for a fair comparison, which presents the percentage of correctly predicted seeds in the test set.

### Feature importance analysis

Linear discriminant analysis (LDA) is a dimensionality reduction and classification technique widely used in supervised learning. In the context of classification, it seeks to discover a linear combination of features that maximizes class or category separation within a dataset. By leveraging dimensionality reduction to enhance classification, LDA identifies the most discriminative features in the dataset (Siqueira et al. 2017; Kaznowska et al. 2018).

Importance of the predictors for classification was obtained by running MATLAB’s built-in-function, *fitcdiscr*, that yields the DeltaPredictor property. Usually, the ’Delta’ property is set to provide a threshold for which predictors to use or not. At the same time, the most informative bands of the hyperspectral data cube were selected by using orthogonal space projection method (Du and Yang 2008).

16 $$\begin{aligned} \text{Y} = \text{X} - \text{P}_{B}{(X)} \end{aligned}$$

where $Y$ is the orthogonal space to subspace $B$, $X$ is the original data matrix, and $P_B(X)$ is the projection of $X$ onto subspace $B$. In MATLAB’s built-in-function *selectBands*, only 10% of the pixel values in the hyperspectral data cube is computed to reduce the computational complexity.

### GWAS analysis

A Genome-Wide Association Study (GWAS) analysis was conducted to explore the relationship between genetic variations and phenotypic traits in KSCC accessions. A total of 420 KSCC accessions were analyzed, with phenotypic data representing 23 features, including seed size, shape, color, endmember abundance proportion, spectral indices, as well as chlorophyll and anthocyanin contents. Genotypic data, comprising of 169,029 Single Nucleotide Polymorphisms (SNPs), were obtained from the 180K Axiom SoySNP Array (Lee et al. 2015; Jeong et al. 2019).

The GWAS analysis employed the Mixed Linear Model (MLM) algorithm implemented in the GAPIT tool (https://zzlab.net/GAPIT/) (Lipka et al. 2012). SNPs with a False Discovery Rate (FDR) threshold of 0.05 or lower were considered significant, while those with a Minor Allele Frequency (MAF) below 5% were excluded from further analysis. Gene information associated with the identified SNPs was obtained from the genetic annotation database, Soybase DB (https://www.soybase.org/). Relevant genes were then subjected to enrichment analysis using the Gene Ontology (GO) analysis tool accessible in the web version of the Soybean Genome Database (SoybeanGDB, https://venyao.xyz/soybeangdb/) (Li et al. 2023).

### Anthocyanin and chlorophyll content

To analyze anthocyanin content, five beans were individually placed in separate tubes and incubated overnight in 300 $\mu$L of methanol acidified with 1% HCl. Following the addition of 200 $\mu$L of distilled water, anthocyanins were separated from chlorophyll by the addition of 500 $\mu$L of chloroform. The concentration of total anthocyanins was determined by spectrophotometric measurements at absorbance values of 530 nm (A530) and 657 nm (A657) in the aqueous phase. The relative anthocyanin content per gram (g) was calculated by subtracting the A657 absorbance from the A530 absorbance (Neff and Chory 1998).

To analyze chlorophyll content, tubes containing a single bean from a set of five were filled with 1 mL of N,N’-dimethylformamide. The samples were incubated overnight to ensure complete chlorophyll extraction. Absorbance at 647 nm and 664 nm was measured using a spectrophotometer. Chlorophyll content was calculated according to the formula by Porra et al. (1989), and then normalized to the weight of the beans.

## Results

### Classification of morphological and color traits of seeds using manual inspection

**Fig. 2:**
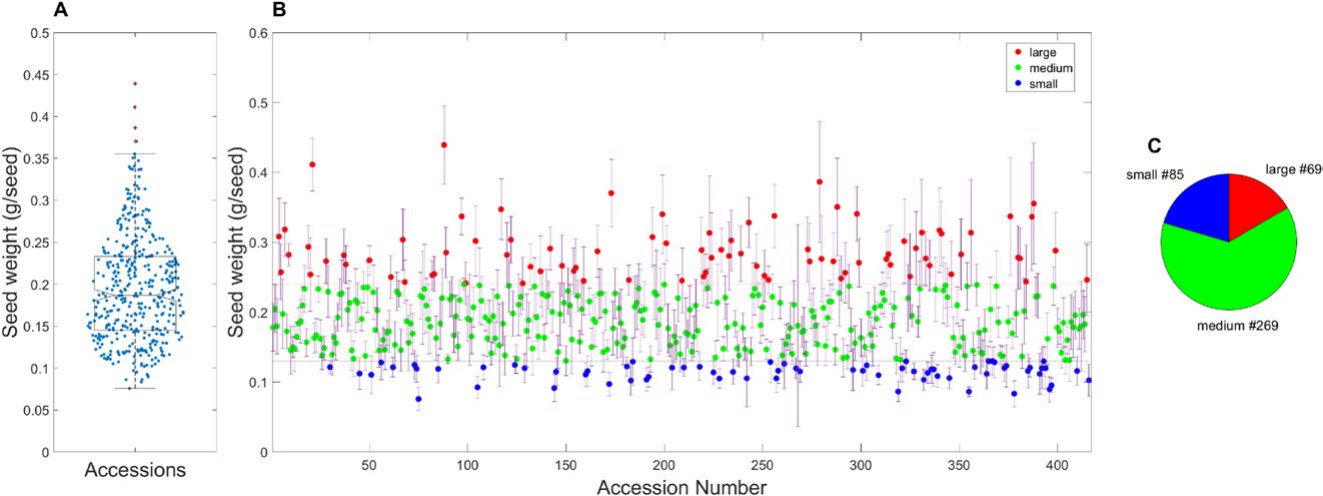
Distribution of seed weight (g/seed, **A**), classification of KSCC accession seeds into small, medium, and large categories (**B**), and count of accessions belonging to each respective size group (**C**) following the UPOV guideline

In total, 420 soybean germplasms were classified based on seed weight, seed shape and coat colors using the criteria provided by UPOV. The single seed weight ranged from 0.0575 to 0.439 g, with a mean of 0.194 g (± 0.063, n = 420) (Fig. 2A). These values are quite similar to soybean germplasms reported earlier (Kim et al. 2022). The lowest and the highest seed weights were observed in accession no. cmj078 and cmj088, respectively. When accessions were classified based on UPOV guideline, the number of accessions belong to small (< 0.13 g), medium (0.13–0.24 g), and large (> 0.24 g) were 85, 262, and 69, respectively, showing respective average weights of 0.11 (±0.02), 0.19 (±0.03), and 0.29 (±0.05) g (Fig. 2B and C).

**Fig. 3:**
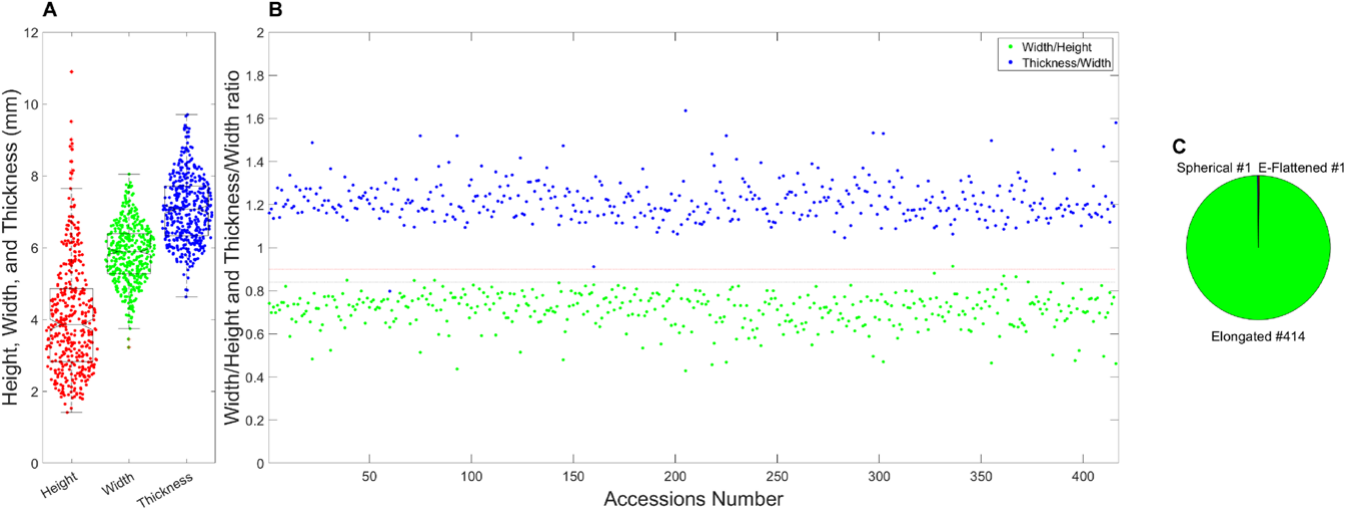
Distribution of seed height, width, and thickness (**A**), ratios of seed width/height and thickness/width (**B**), and count of KSCC accessions belonging to each respective seed shape (**C**) following the UPOV guideline

To determine seed shape, RGB images were generated from the HSI, and then ImageJ software was used to measure area, major- and minor-axis lengths for height, width, and thickness, respectively. Seed height ranged from 1.42 to 10.89 mm, with a mean of 4.08 mm (± 1.59) (Fig. 3A). Seed width ranged from 3.23 to 8.05 mm, with a mean of 5.85 mm (± 0.83) (Fig. 3A). Seed thickness ranged from 4.63 to 9.71 mm, with a mean of 7.08 mm (± 0.93) (Fig. 3A). For the classification of seed shape based on UPOV guideline (i.e., spherical (Width/Length > 0.90 and Thickness/Width > 0.85), spherical-flattened (Width/Length > 0.90 and Thickness/Width > 0.84), elongated (Width/Length < 0.89 and Thickness/Width > 0.85), and elongated-flattened (Width/Length < 0.89 and Thickness/Width < 0.84), 414 seeds belong to the category no. 3 (elongated), and the remaining accessions, cmj063 and cmj348, belong to the spherical and elongated-flattened, respectively (Fig. 3B and C). No flattened (Thickness/Width ratio $\le$ 0.84) germplasm was found.

**Fig. 4:**
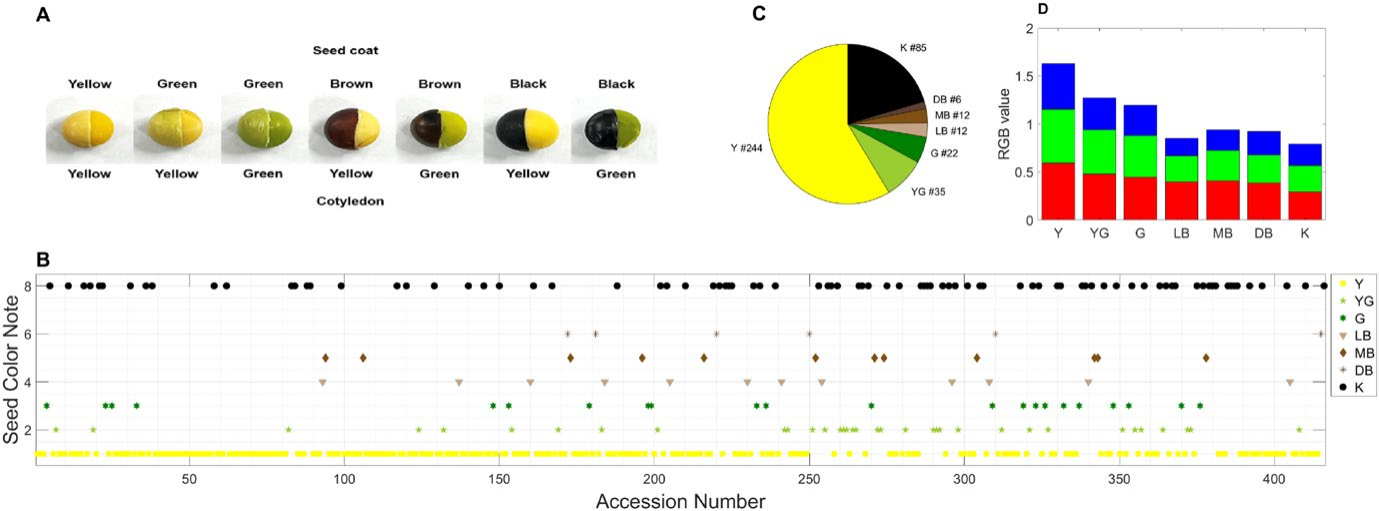
Images of KSCC accession seeds showing seed coat and cotyledon colors (**A**), a scatter plot illustrating seed coat color categories (**B**), the count of KSCC accessions belonging to seed colors - yellow (Y), yellow-green (YG), green (G), light brown (LB), medium brown (MB), dark brown (DB), and black (K) (**C**) - according to the UPOV guideline, and averaged RGB triplets for each respective seed color (**D**)

The seed coat colors of soybeans are diverse including yellow, green, brown and black (Baek et al. 2020; Kim et al. 2022). In comparison, only two colors, yellow and green observed in cotyledons (Fig. 4A). Through visual inspection, the seed coat colors of 420 accessions were classified into seven different categories: (1) yellow, (2) yellow green, (3) green, (4) light brown, (5) medium brown, (6) dark brown, and (8) black, as shown in Fig. 4B. Purple-colored seed accessions were not found in the KSCC germplasm, as previously reported from the other source of soybean germplasm (Kim et al. 2022). Yellow (n = 244) was the most frequent classification, followed by black (n = 85), yellow-green (n=35), green(n = 22), light brown (n = 12), medium brown (n = 12), and dark brown (n = 6) (Fig. 4B and C). For bi- and multiple-color seed coats such as black with white stripes or dots and brown with white stripes or dots, the colors were classified as black or brown, respectively. However, the averaged RGB triplet and grey scale of medium- and dark-brown seed coats were [0.41 0.32 0.26 0.30] and [0.39 0.29 0.24 0.31], being statistically not different (*p* < 0.5, Fig. 4D). Therefore, we propose that KSCC accessions are now classified as 6 categories, i.e., yellow, yellow green, green, light brown, brown, and dark.

### Seed endmembers extraction and seed segmentation

**Fig. 5:**
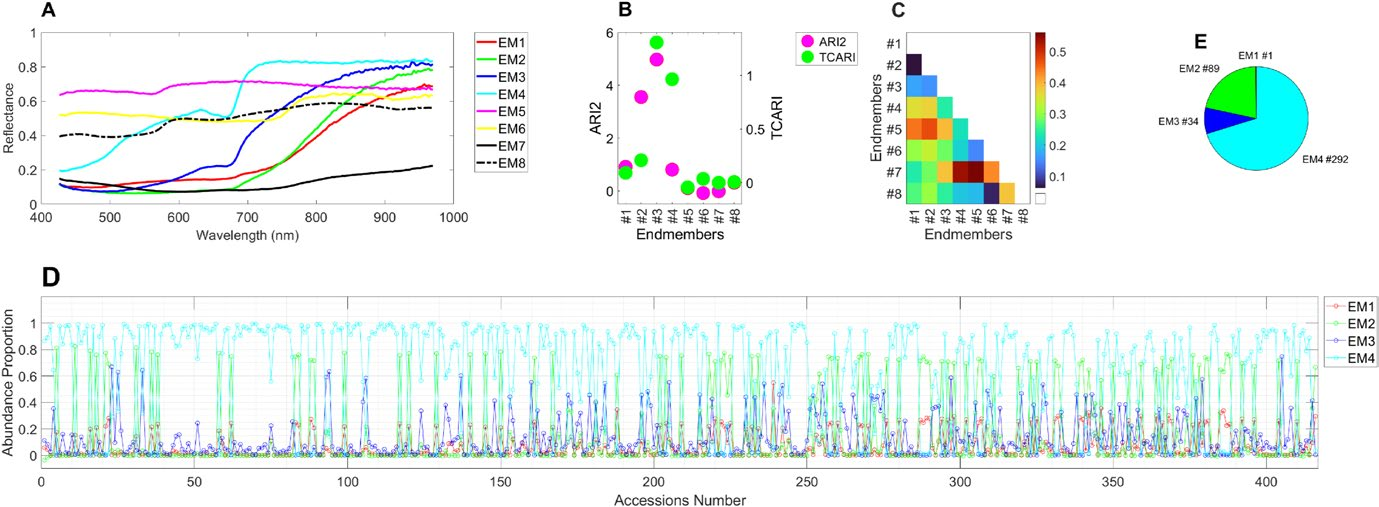
Reflectance spectra of endmembers extracted from KSCC accession seeds (**A**), vegetation indices for anthocyanin (ARI2) and chlorophyll (TCARI) (**B**), NS3 similarity values among endmembers (**C**), abundance proportions of foreground seeds targeted endmembers for ventral seed sides (**D**) and the count of KSCC accession seeds with the most abundant endmembers (**E**)

Seed segmentation using RGB triplet codes based on global or multiple thresholds in the current study was not successful, resulting in low accuracy (number of seeds segmented / number of seeds imaged x 100) for separating seed images from the background images as well as neighboring seeds (4.3%). This low accuracy was mainly caused by shadows that overlapped with neighboring seeds, making it challenging to separate individual seeds. Therefore, we aimed to filter out shadow pixels using spectral unmixing technology. Additionally, pixels are not pure; instead, they are subject to mixed pixel issues (Sanjeevi and Barnsley 2000), which involve the mixture of multiple pure endmembers. Thus, spectral unmixing was applied to extract endmembers and quantify their abundance proportion. In the present study, mixed pixels were unmixed using the linear mixing model, PPI algorithm. Out of 420 HSI of seed accessions, a total of 2,567 endmembers were initially isolated, with a mean of 6.17 (± 1.84) endmembers per accession. After filtering out non-redundant endmembers based on the mean-squared error (MSE) among endmembers using a cutoff value of 5 x $10^{-5}$, 8 endmembers were finally selected (Fig. 5A). Unmixing accuracy evaluated using rRMSE was 2.12% (± 0.37, n = 420). Furthermore, seed segmentation accuracy was 98.8%, significantly enhanced compared to that based on RGB images.

Based on visual inspection of abundance maps (Suppl. Fig. S1), these endmembers could be assigned to either the foreground target (named hereafter as EM#1, 2, 3, and 4) or background pixels (named hereafter as EM#5, 6, 7, and 8). The first group endmembers are refined to the seed coat and hilum, while the 2nd group mostly represents background pixels such as shadows and white cloth (Suppl. Fig. S1). Vegetation indices of these endmembers, such as ARI2 index for anthocyanin pigment (Gitelson et al. 2003) and TOCARI index for chlorophyll pigment (Haboudane et al. 2004) (Fig. 5B), were different among endmembers. Index values of foregrounds showed greater values than those of the background. Roughly speaking, endmembers #1, 2, and 4, and 3 are correlated to respective yellow, brown and green, and black seed colors.

Next, we carried out spectral similarity analysis between test spectra and the specified reference spectrum using SAM classification algorithm (Kruse et al. 1993), SID technique (Chang 2000), and NS3 (Nidamanuri and Zbell 2011), respectively. As shown in Fig. 5C and Suppl. Fig. S2A and B, spectral dissimilarity was found within foreground endmembers as well as between those of foregrounds and backgrounds. Figure 5D and E and Supplementary Fig. S2C and D show abundance proportions of endmembers for the ventral and lateral sides of seeds, respectively. In both side images, EM#4 (n = 292 for ventral side, and 275 for lateral side) was the most abundant endmember, followed by EM#2 (n = 88 and 89), EM#3 (n = 52 and 34), and EM#1 (n = 1 for both). Here, the endmember proportion in the seed image represents the abundances of a given endmember unmixed from the other target endmember classes. For instance, a proportion value of 0.80 from the abundance image indicates that 80% of the target consists of seed testa classified by a given endmember. For both ventral and lateral sides, EM#1 was the most abundant endmember, and hence likely contributed to distinguish seed coat from the other parts. For the case of EM#1 abundance value is > 0.80, the hilum’s spectral property seems very similar to that of seed coat.

### Correlation between seed matrices acquired through **SUnSet** and those obtained manually

To evaluate the effectiveness of the algorithm on seed segmentation with different morphological traits, morphological statistics such as vertical area, seed height, width, and thickness were compared with those obtained from ImageJ. A total of 5,382 seeds from 420 accessions, with a mean of 12.93 (± 0.58, no.of seeds/accession), were measured and compared in terms of area, height, and width. The correlation coefficient values ($r^2$) of these traits between seeds segmented from the hyperspectral unmixing algorithm and ImageJ were 0.98, 0.94, and 0.93, respectively, indicating that the size and shape seeds are accurately segmented by **SUnSet** toolbox.

### Spectral analysis

**Fig. 6:**
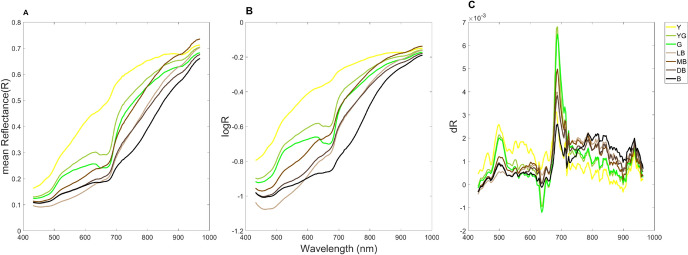
Averaged relfectances (**A**), and their logarithmic transformations (**B**) and first derivatives (**C**) of KSCC accession seeds, grouped by seed coat colors - yellow (Y), yellow-green (YG), green (G), light brown(LB), medium brown (MB), dark brown (DB), and black (K) (color figure online)

The averaged hyperspectral reflectance of seed accessions was obtained by averaging the reflectance of segmented seeds. As shown in Suppl. Fig. S3, each curve represents the averaged reflectance of approximately 50 seeds in the corresponding group. The average reflectance curves showed great diversity, including similar patterns of vegetation with the red-edge region, which is the position of the main inflection point of the red-NIR slope (670–780 nm) (Curran et al. 1995; Van Der Meer 2004). At the same time, stronger or weaker red-edge inflections were also observed. It is known that strong chlorophyll absorption in the red spectrum and scattering in the NIR cause the Red-Edge Position (REP) phenomenon (Dawson and Curran 1998). The differences in the spectral response in the visible light region are indicative of seed pigments. For instance, seeds with green colors would consequently have higher reflectance within the green reflectance spectral region. Consistently, the averaged hyperspectral reflectance of yellow, yellow-green, and green seed colors show greater inflection in the green light region, while this inflection is the lowest in the light brown seed coat group (Fig. 6A).

Logarithmic (logR; Fig. 6B) and first derivative (dR; Fig. 6C) transformations also clearly yielded spectral variations among different seed coat color groups. Furthermore, the seeds from the KSCC collection were subjected to additional categorization through hierarchical clustering. Spectral similarity among the averaged reflectance of KSCC accession seeds were assessed using SAM, SID, and NS3 algorithms. Subsequently, linkage and cluster functions were applied to group these spectra based on their similarity values obtained from pairwise comparisons. As a result, the KSCC accession seeds were grouped into 20, 25, and 27 subclusters (Suppl. Fig. S3), highlighting the potential of hyperspectral reflectance as a predictive factor for seed classification when employing machine learning classifiers.

### Supervised learning based classification of KSCC seeds

To determine seed accessions using machine learning classifiers (Zhang et al. 2018; Zhu et al. 2021), we utilized 199 predictors, including morphological features such as seed weight, shape, and size (as shown in Figs. 2, 3, and 4), chlorophyll and anthocyanin pigment contetns, RGB triplets, and hyperspectra-derived features. Hyperspectral features comprised the averaged reflectance of each seed coat and hilum segmented by endmembers (Figs. 5 and 6), along with their log transformation (logR) and 1st derivatives (dR), as well as vegetation indices for anthocyanin and chlorophyll contents. For RGB and hyperspectral features, each band or wavelength was considered a feature. The response classes consisted of 420 KSCC accession seeds and the number of training samples matched the total number of seeds. A total of 19,933 or 19,934 seeds were randomly split into training and test samples–15,947 or 15,948 for training and 3,986 for testing–using four different random number generator algorithms, i.e., twister (mt19937ar)(Marsaglia and Tsang 2000), combRecursive (mrg32k3a) (L’Ecuyer 1999), multFibonacci (mlfg6331_64) (Mascagni and Srinivasan 2004) and philox (philox4x32_10) (Salmon et al. 2011).

**Fig. 7:**
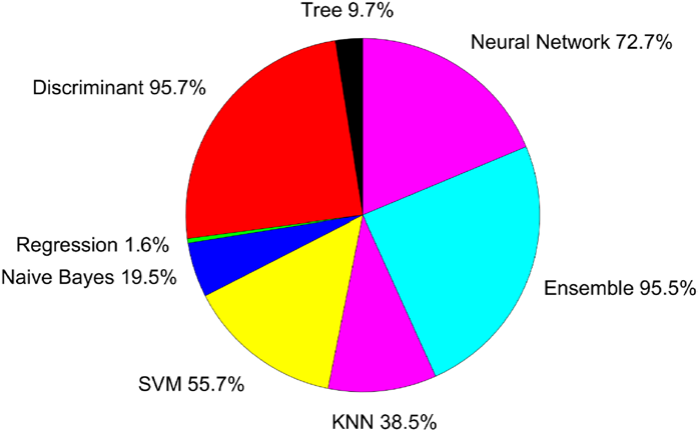
Classification accuracy scores for the eight machine learning models (tree, discriminant, regression, Naive Bayes, support vector machine (SVM), k-nearest neighbor (KNN), ensemble and neural network (NN)), based on the datasets containing all features

The model’s accuracy in classifying seeds into their respective accession numbers on the training samples varied in the range of 9.7% to 95.8%, with the highest accuracies observed for LDA (95.8%) and its subspace LDA Ensemble (95.4%) (Fig. 7). Prediction accuracies using morphological and RGB features resulted in 24.7%, while hyperspectral features achieved 94.3%, suggesting that prediction accuracy is primarily related to hyperspectral features. Accuracies using logR and dR derivatives of averaged spectra had minimal impact on prediction matrices, obtaining 94.3% and 94.1% prediction accuracies, respectively. In the present study, principle component analysis (PCA)-dependent reduction of spectral bands did not significantly increase prediction accuracy, if at all. Metrics for LDA classification are shown in Table 1.

**Table 1:** Classification model performance assessment

Model	Kappa	Precision	Recall	F1-score
LDA^1^	1.00	0.975	0.975	0.975
Ensemble^2^	1.00	0.96	0.96	0.96

^1^ Linear Discrimination Analysis

^2^ Subspace LDA Ensemble

### Major wavelengths determining seed classification

**Fig. 8:**
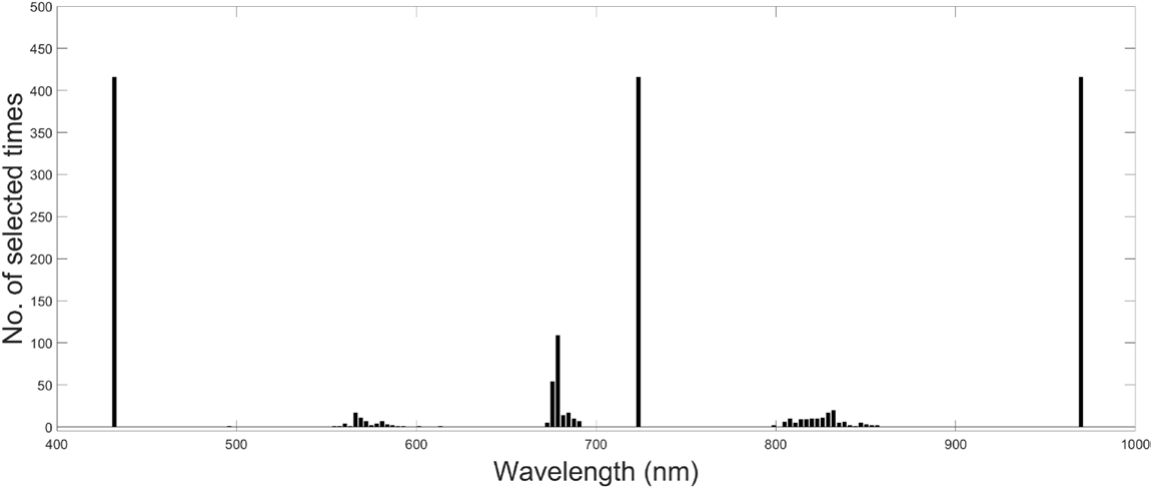
Frequency histogram of the most informative spectral bands (wavelengths) of the hyperspectral data cube obtained using the orthogonal space projection method (Du and Yang 2008). Foreground abundant endmembers EM#1, 2, 3, and 4 extracted by PPI algorithm (Fig. 5A) were used

The extracted spectrum includes 183 wavelengths after filtering out both end bands. To identify the bands that are determining features for accurate prediction, bands were selected using Matlab’s built-in function *selectBands*, resulting in 1,664 bands from 420 seed accessions. Out of the 183 bands, the 46 bands exhibited a selection frequency of more than once, whereas the remaining 139 bands were not chosen at all (Fig. 8). This highlights that 25.1% of the wavelengths play a pivotal role in seed classification based on averaged reflectance spectra. Among the selected bands, three bands – no. 1 (corresponding to 427 nm), 100 (721 nm), and 181 (973 nm) – were found in every seed accession. Other bands were mostly located in three regions, centering around green (554 nm), red (672 nm), and NIR (823 nm).

Next, we used the reflectance values of the selected bands as predictors to test if the 46 features significantly contributed to seed classification. The LDA classifier, using the 46 selected features, resulted in an overall accuracy of 62.6% (± 13.08, n = 4), which is higher than those obtained by morphological and RGB features but lower than those of full spectral features.

### GWAS analysis using hyperspectral features reveals genes regulating seed shape, size and pigmentation

**Fig. 9:**
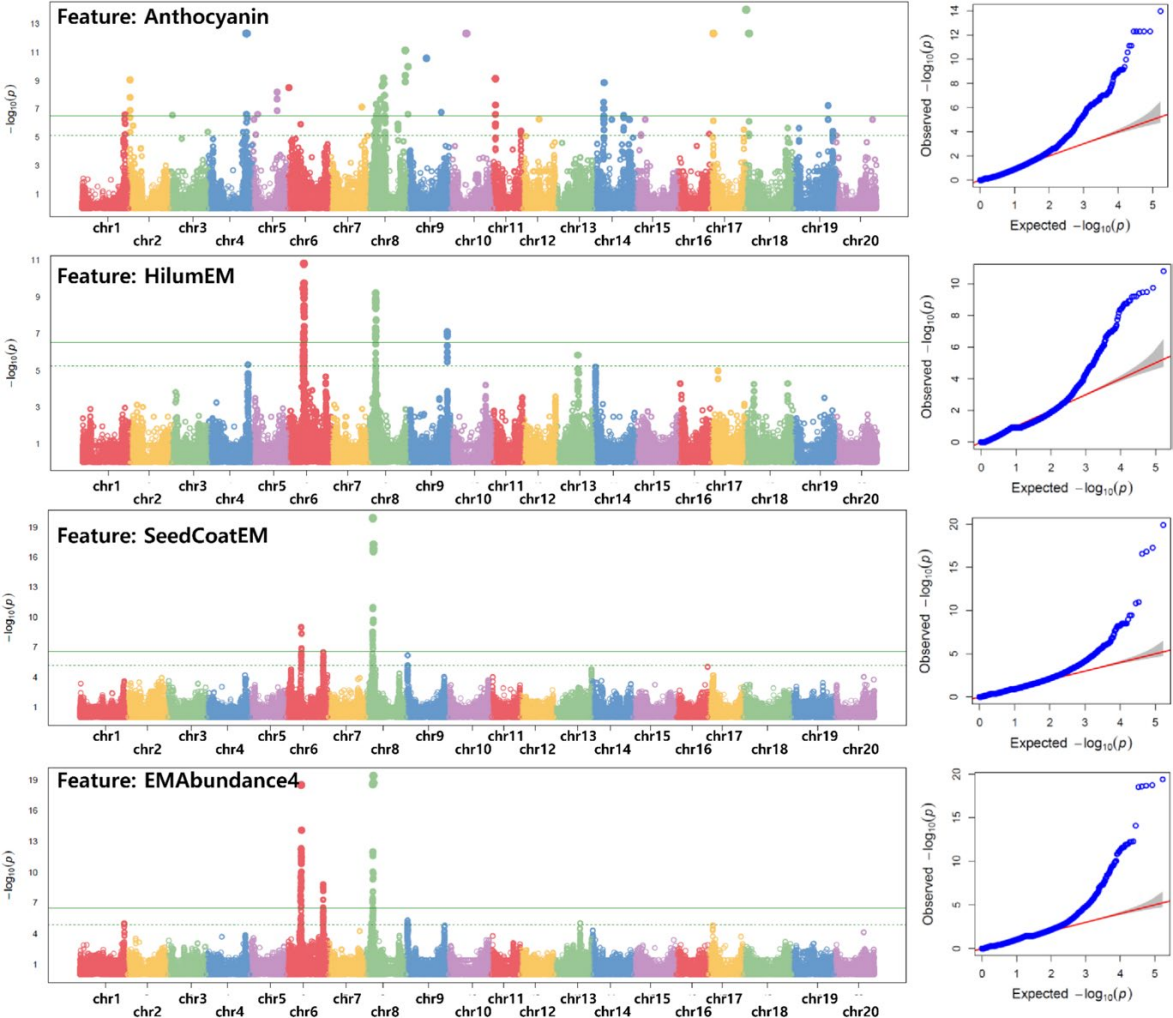
Manhattan (left) and Quantile-Quantile (right) plots of the GWAS generated by GAPIT to identify significant associations between SNPs and hyperspectral features for anthocyanin, hilum and seed coat abundant endmembers, and abundance proportion of endmember EM#4. The dashed horizontal line represents the genome-wise significance threshold of 5 where the (-log P) values of the range from 1.1 to 20

We processed to assess whether features extracted from HSI analysis using **SUnSet** accurately represent KSCC accession seed traits. Therefore, GWAS analysis was conducted to extract and validate these endmember features associated with seed traits, followed by establishing their correlation with actual soybean traits (Fig. 9). In the present study, features obtained from the **SUnSet** toolbox include seed shape, represented by the ratio of thickness/width and length/width, seed coat and hilum colors grouped visually or by their RGB triplet codes and grayscale values obtained from their respective color groups, chlorophyll and anthocyanin pigment contents, endmembers abundance proportions, and spectral indices of vegetation (NDVI), chlorophyll (TCARI), and anthocyanin (ARI2) from both the ventral and lateral sides of the seed (Supplementary Table S1).

With an FDR threshold of < 0.05 and MAF 5%, GWAS analysis revealed significant SNP associations for 23 features used in the machine learning LDA analysis. Specifically, associations were identified for 191 SNPs with seed shape, 145 with anthocyanin contents, 5 with seed coat color category, 225 with seed coat RGB code and grayscale, 962 with seed coat and hilum abundant endmembers and their proportionality, and spectral indices, resulting in a total of 1,615 SNPs (Supplementary Table S2 and Table 2). Chromosome regions displaying significant associations with seed coat and hilum colors were identified on Chromosomes 1, 6, 8, 9, and 20 (Fig. 9, Supplementary Fig. S4 and Table 3), consistent with previous research findings (Sonah et al. 2015; Vuong et al. 2015; Zhou et al. 2015; Fang et al. 2017). Additionally, 13 additional regions related to seed color and 4 regions related to seed shape were newly identified on Chromosomes 4, 6, 7, 8, 14, 16, 17, 18, and 19, as well as Chromosomes 6, 7, 8, and 18, respectively. Notably, four regions simultaneously associated with both seed shape and color were discovered on Chromosomes 6 and 8.

**Table 2:** Summary of GWAS analysis results

Features	SNPs	Genes^1^	Intergenic Region
*Seed shape*			
Thickness/Width ratio	172	96, 68	76
Width/Length ratio	19	15, 9	4
*Pigments*			
Anthocyanin	93	47, 37	46
Chl	52	23, 20	29
*Seed Coat color*			
visual	1	1, 1	0
predicted Gray	4	4, 2	2
*Seed coat color code*			
Red	88	50, 44	38
Blue	22	15, 10	7
Green	44	26, 18	18
Gray	71	42, 31	29
*Ventral side endmember abundance*			
EM#1	130	68, 48	62
EM#2	192	104, 72	88
EM#3	17	9, 6	8
EM#4	299	141, 91	158
*Abundant endmember*			
Hilum	154	57, 40	97
SeedCoat	138	73, 47	65
*Lateral side endmember abundance*			
EM#1	32	11, 11	21
*Ventral side VI*			
NDVI	8	3, 3	5
TCARI	3	2, 2	1
ARI2	32	13, 10	19
*Lateral side VI*			
NDVI	8	5, 4	3
TCARI	4	2, 2	2
ARI2	32	13, 10	19

^1^The number of genes mapped with single or multiple SNPs is provided as a pair of values separated by a comma

Consistently, among 229 genes associated with these SNPs (Supplementary Table S3), genes associated with the phenylpropanoid pathway are prevalent (Supplementary Table S4), suggesting a significant correlation between the **SUnSet**-derived input phenotype features and the identified genes. Furthermore, quantitative trait loci (QTLs) linked to seed coat and hilum traits are located on chromosome 8 in cultivated soybean recombinant inbred lines (RILs) and natural populations (Yang et al. 2023), emphasizing its relevance in this study.

**Table 3:** Chromosome regions displaying significant associations with seed color and shape traits either independently or collectively

Traits/features	Chr/Significant regions			References
*Regions consistent with findings from prior studies*				
**Hilum color**				
Chl, EM#1,#2,#4, Red, Gray, SeedCoat, Green, NDVI(V), EM#1	Chr06	13,675,166–19,332,093		Vuong et al.^2015^
Anthocyanin	Chr08	12,106,211–12,616,101		Fang et al. (2017); Sonah et al. (2015)
Chl, EM#1, 2 and 4, TCARI	Chr09	41,379,836–46,522,279		Sonah et al. 2015
Chl	Chr20	33,596,377–33,605,144		Fang et al. (2017)
**Seed coat color**				
Anthocyanin, EM#3, EM#4, Red, ARI2	Chr01	49,844,155–53,475,445		Vuong et al. (2015); Fang et al. (2017)
Chl, EM#1,#2, and #4, Red, Gray, SeedCoat, Green, NDVI, EM#1	Chr06	13,675,166–19,332,093		Vuong et al. (2015)
Anthocyanin, EM#1,#2, and #4, Red, Green, Gray, SeedCoat, Hilum, NDVI	Chr08	6,481,637-11,394,472		Zhou et al. (2015); Vuong et al. (2015)
**Traits/Features**	**Chr./Significant regions**			
*New regions identified in the current study*				
**Chl**				
Hilum	Chr04, 46,587,701-46,807,590; Chr16, 35,654,165–35,810,584			
EM#1, 2 and 4, SeedCoat, Red, Gray, Green	Chr06, 44,539,514–47,802,858; Chr17, 5,311,321-5,405,549			
**Thickness/width ratio**				
EM#2, SeedCoat	Chr06,6,752,891–6,827,909; Chr17, 1,540,064–4,358,709			
NDVI(L)	Chr08, 5,096,452–5,109,650; Chr18,38,015,496–56,429,249			
**Hilum color**				
Chl	Chr07, 43,878,518–44,021,098; Chr17, 8,533,636–8,578,296			
**Anthocyanin**				
Chl	Chr08, 279,247–288,750; 14,707,723–15,072,254;			
	Chr17, 39,921,437–39,927,901			

## Discussion

The proposed **SUnSet** toolbox offers a comprehensive solution for hyperspectral imaging of seeds, covering the entire process from acquisition to analysis. Unlike previous approaches that predominantly rely on analyzing mixed pixels, this system is specifically based on a linear unmixing algorithm for hyperspectral imaging analysis. As a result, it has demonstrated high segmentation and classification accuracies for seeds across 420 different accessions. Furthermore, it has identified several candidate chromosome regions that are potentially associated with seed shape determination and the coloration of seed coat and hilum.

Seed size has mostly been classified based on the 100-seed weight (UPOV guideline), hinting that 2D RGB image analysis makes it hard to assess this 3D parameter. Contrary to this assumption, seed area, height and width on the vertical side quantified by **SUnSet** or ImageJ software resulted in high linear correlation to the seed size ($r^2$ = 0.92  ~  0.98). However, $r^2$ value was lower when seed thickness were compared with seed weight ($r^2$ = 0.68). Thus, the ventral area, height and width of the seed seems highly likely to represent seed weight.

Seed coat or hilum color has been classified by trained visual inspection, which is quite qualitative and subject to variations as the apparent color is a result of the combination of genetic and environmental factors. So far, RGB codes have been assigned only to the representative soybean core collections (Baek et al. 2020; Kim et al. 2022), but those for every core collection accession have not been attempted on a whole-core collection scale. Averaged RGB triplets for seven seed coat colors obtained in the present study revealed medium and dark brown seed coats are statistically indistinguishable. However, averaged reflectance spectra of seeds, and their log and 1st derivative transformants clearly distinguish two colors due to differences in the green and red bands along with the NIR region (Fig. 6). Thus, RGB triplet codes obtained in the present study could be used as reference values for classifying RGB- or hyperspectral-images of soybean seeds specifically and other crop seeds with diverse genetic diversity in seed colors in general.

Spectral unmixing done in the present study yielded four target seed endmembers and their seed coat- and hilum-abundance maps (Fig. 5 and Suppl. Fig. 1), which is practically hard to obtain manually. By simply multiplying abundance maps and ventral areas, seed coat and hilum sizes are easily obtainable. Therefore, spectral unmixing could be a useful analysis tool to assess seed coat and hilum size. Of course, seeds with either seed coat and hilum are indistinguishable visually or striped- or dotted-coat seeds; this way of image segmentation is not applicable.

Morphological functions using grey or color thresholders are widely used in seed segmentation for both RGB and HSI, but seed segmentation accuracies in the present study are lower than 25%, hampering further image analysis. This lower accuracy was significantly enhanced up to 99.8% by the directional gradient of gray images that are targeted to the endmembers-selected foregrounds. Thus, spectral unmixing turns out to be a critical step for seed image segmentation and hence reliable extraction method of seed morphological, color, and spectral features. Indeed, morphological traits such as area, width, and height of seeds obtained based on the current study almost perfectly match those from ImageJ software.

The classification accuracy of KSCC seeds is best performed by LDA, a dimensionality reduction and classification technique commonly used in machine learning. LDA aims to reduce the dimensionality of the feature space while retaining as much class-separability information as possible. It has proven successful in discriminating between *Fusarium* damaged wheat kernels and undamaged ones (Delwiche et al. 2011). In the domain of seed classification using LDA, hyperspectral features have shown superior performance compared to morphological and RGB color features. This improved performance is likely attributed to the inclusion of features in the near-infrared (NIR) range, which are absent in RGB-based features. Indeed, NIR bands were among the 46 selected bands in addition to green and red bands (Fig. 8). Similarly, in the classification of red- and white-wheat kernels, reflectance primarily from O-H and C-H bands in the short-wave (SW) NIR range (700 - 1,100 nm) plays a crucial role in predicting the color class of grains (Archibald et al. 1999).

Spectral transformations and vegetation indices play a crucial role in mitigating the impact of brightness variations on ground-based spectral measurements. Previous studies have demonstrated the effectiveness of classification performance using first derivative and log-transformed datasets, as well as selected hyperspectral vegetation indices, in discriminating between seed and vegetation species (Polder et al. 2002; Gao et al. 2021). However, in the present study, despite utilizing log transformation and first derivative spectra for classification, the accuracy of LDA classification showed minimal improvement. Additionally, classification accuracies using features derived from chlorophyll and anthocyanin indices were even lower than those obtained using morphological features. This disparity could be attributed to the successful filtering out of brightness variations during the spectral unmixing process. Consequently, spectral unmixing of hyperspectral imaging proves to be effective for soybean core collection seed identification, with LDA serving as an efficient classification technique.

The integration of large-scale hyperspectral data into GWAS has emerged as a potent strategy for elucidating the genetic foundations of agricultural traits and expediting crop enhancement (Feng et al. 2017; Sun et al. 2019; Barnaby et al. 2020; Wu et al. 2021; Massahiro Yassue et al. 2023). In our present investigation, we reinforce this perspective by employing the **SUnSet** toolbox. As a result, the discerned broad-spectrum endmember features exhibit considerable efficacy in pinpointing valuable genetic loci in soybeans. This alignment with known anthocyanin Quantitative Trait Locus (QTL) regions on chromosome 8 underscores the significance of our findings and bolsters the effectiveness of the broad-spectrum endmember features identified in our study. Moreover, beyond the identification of genes related to phenylpropanoid metabolism, several candidate genes implicated in regulating seed size, shape, and colors including auxin signaling and cell expansion, carotenoid, and chlorophyll metabolisms are identified (Supplementary Tables S3 and S5).

## Conclusion

We present an end-to-end software solution named **SUnSet** designed to process HSI of soybean seeds, with applicability to seeds from various plant species. The spectral unmixing step in the software effectively extracts hyperspectral reflectance data for the segmented seeds as a whole, as well as for seed parts such as testa and hilum. The software’s output includes morphological traits based on seeds, RGB color codes, and averaged reflectance data for each seed. The classification of seeds involves the utilization of extracted reflectance data from 420 KSCC accession seeds. Among various machine learning models compared, LDA demonstrated high accuracy in seed classification. Furthermore, the spectral curves of the seeds were meticulously analyzed, leading to the identification of wavelengths with significant importance and genes controlling complex seed trait.

### Supplementary Information

Below is the link to the electronic supplementary material.

Supplementary file 1 (pdf 2181 KB)

Supplementary file 2 (xlsx 458 KB)

## Data Availability

The datasets generated during and/or analyzed during the current study are available from the corresponding author on reasonable request.
