# Supplementary material for: SUnSeT: spectral unmixing of hyperspectral images for phenotyping soybean seed traits
Source: Plant Cell Rep. 2024 Jun 9;43(7):164. doi: 10.1007/s00299-024-03249-0 (PMC11162974; doi:10.1007/s00299-024-03249-0)
Supplement: Supplementary file 1 — (pdf 2181 KB) [file 299_2024_3249_MOESM1_ESM.pdf]

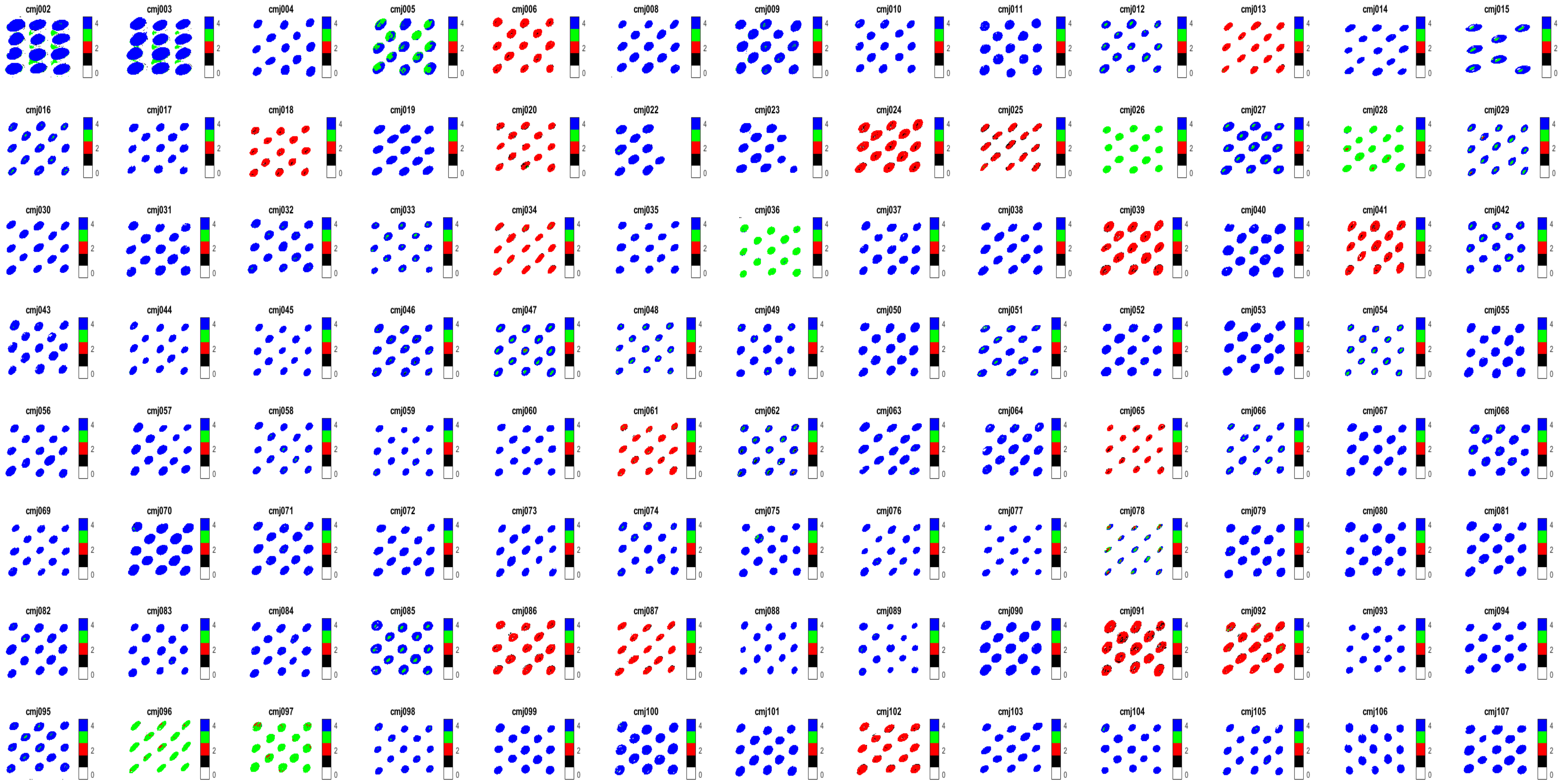

Supplementary Figure S1-1. Abundance maps for endmembers (#1, 2, 3 and 4) of KSCC accession seeds. The maps display 104 accessions from cmj002 to cmj107.

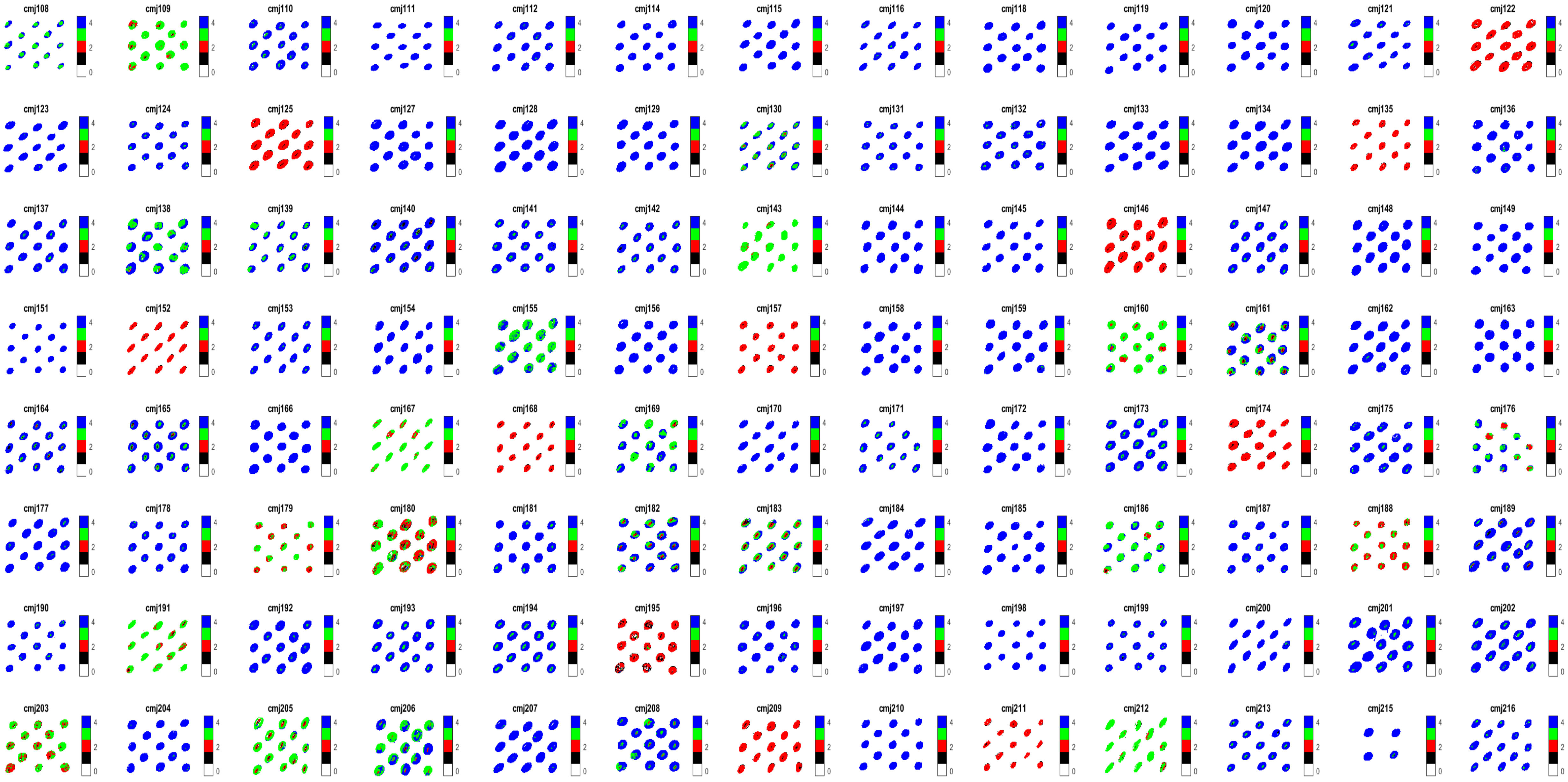

Supplementary Figure S1-2. Abundance maps for endmembers (#1, 2, 3 and 4) of KSCC accession seeds. The maps display 104 accessions from cmj108 to cmj216.

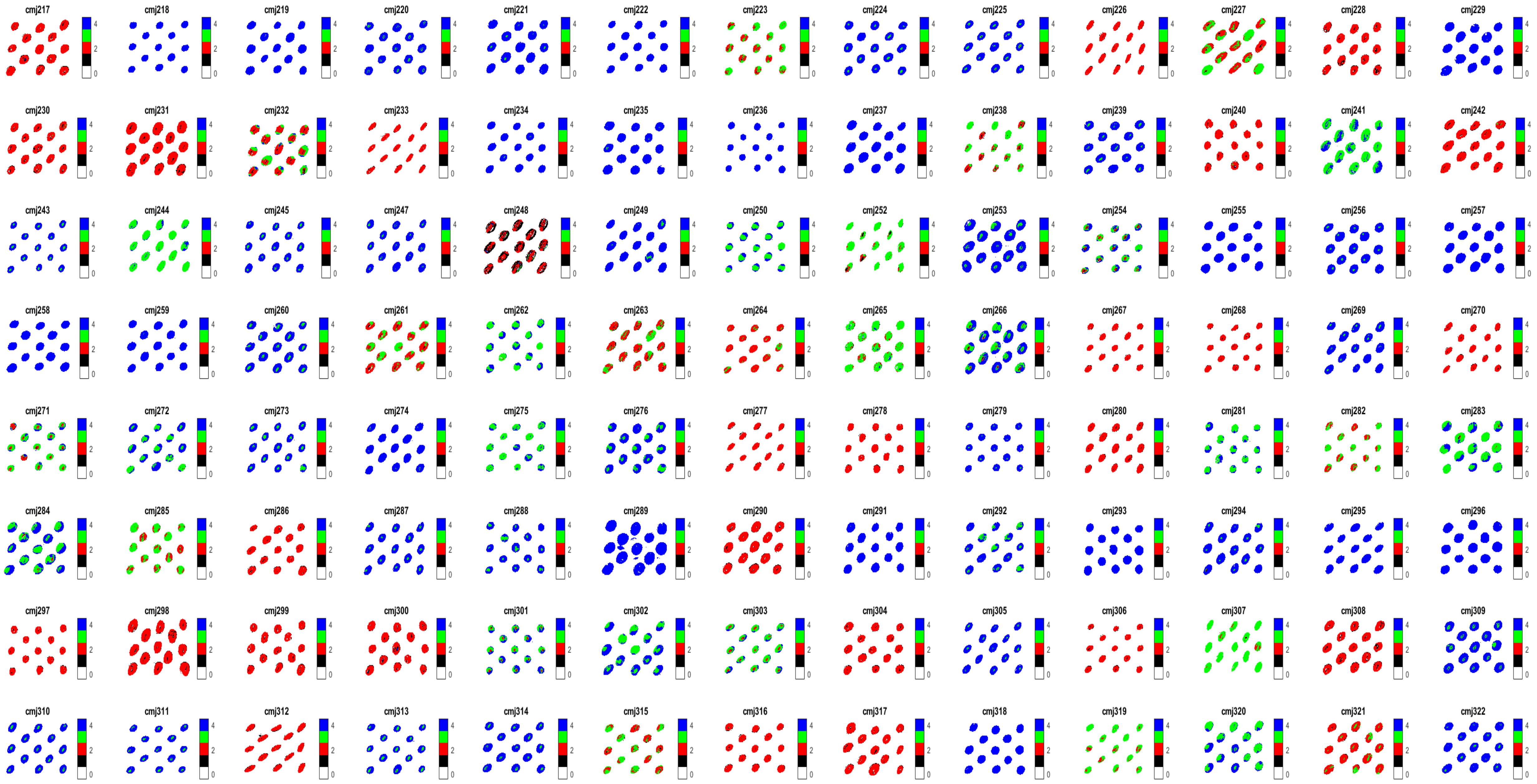

Supplementary Figure S1-3. Abundance maps for endmembers (#1, 2, 3 and 4) of KSCC accession seeds. The maps display 104 accessions from cmj217 to cmj322.

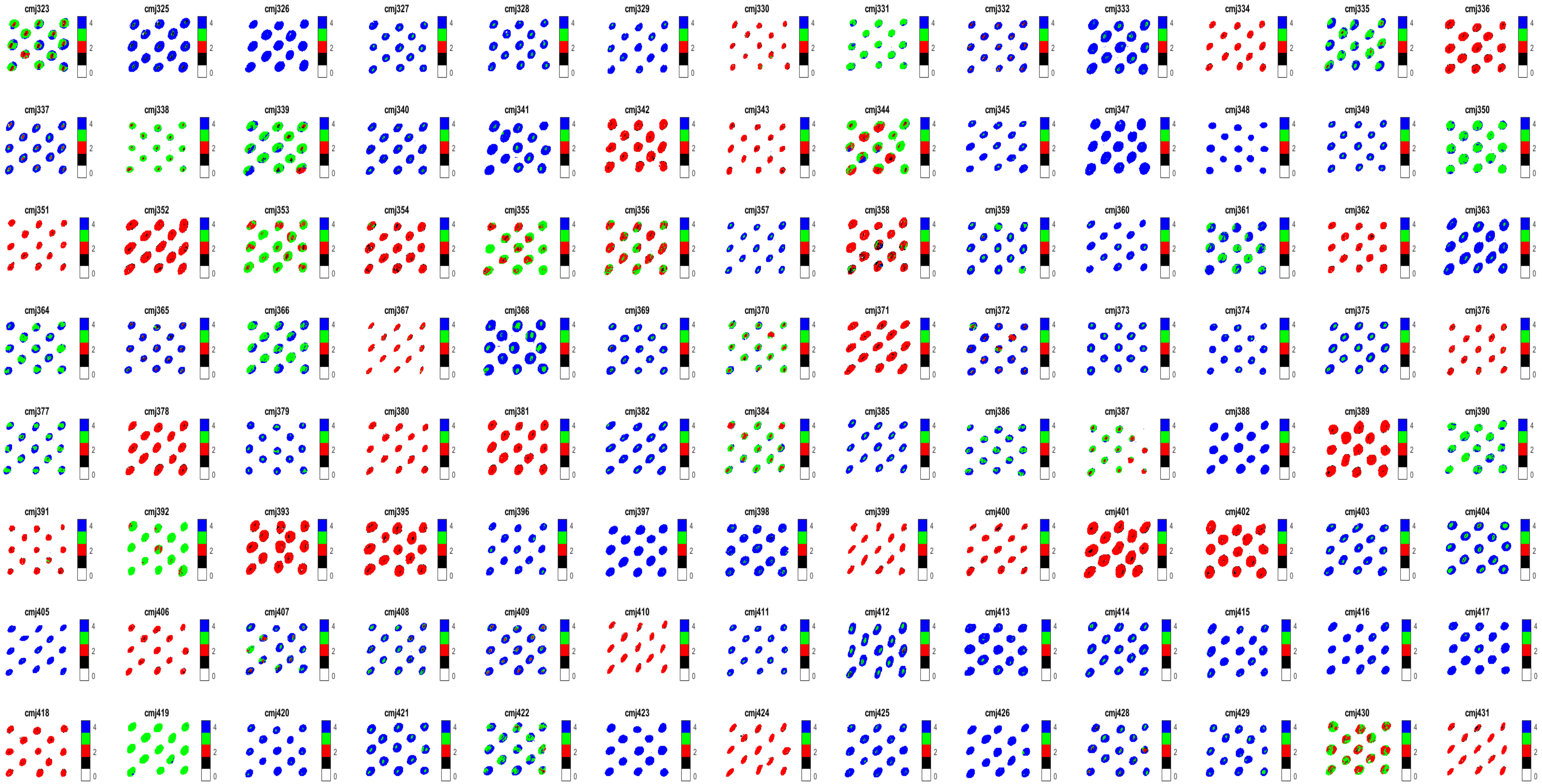

Supplementary Figure S1-4. Abundance maps for endmembers (#1, 2, 3 and 4) of KSCC accession seeds. The maps display 104 accessions from cmj322 to cmj431.

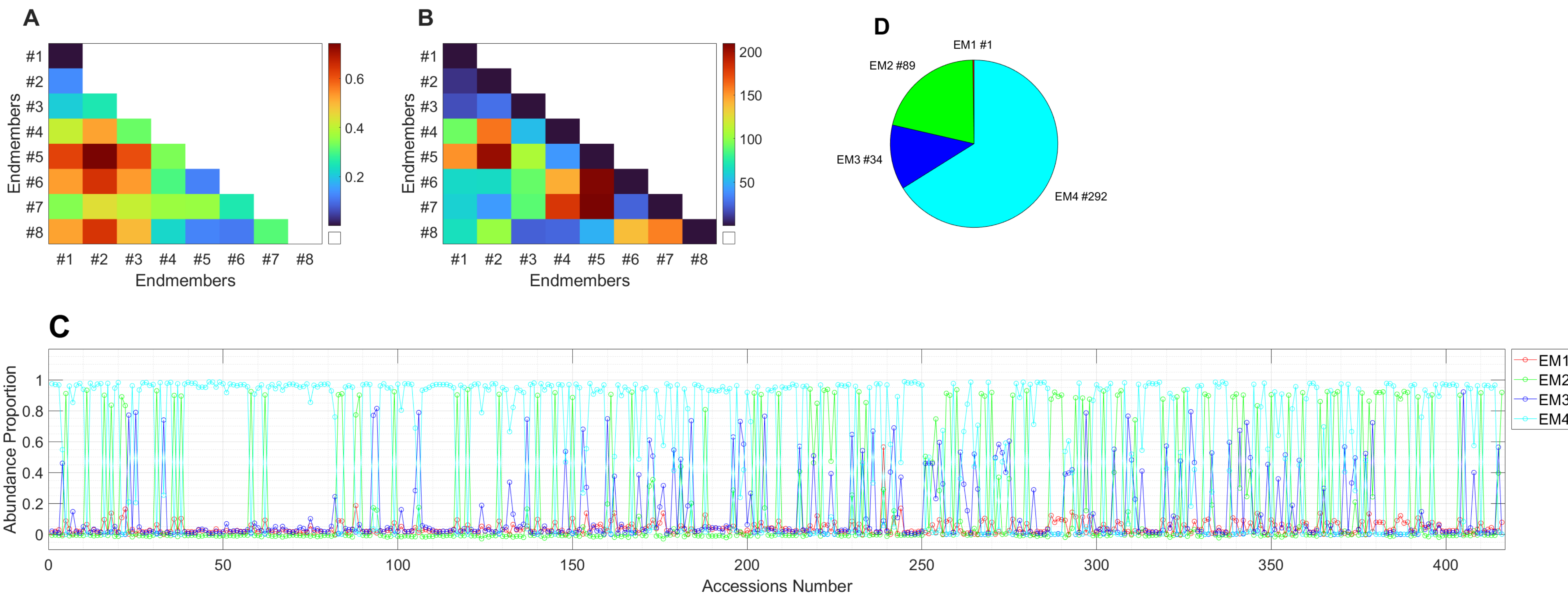

Supplementary Figure S2. Comparison of similarity between endmembers extracted from KSCC accession seeds (A, B) and abundance maps (C), along with the count of endmembers showing the most abundant proportion in given accession seed (D). In panels A and B, SAM and SID algorithms were employed to calculate similarities among endmembers, respectively

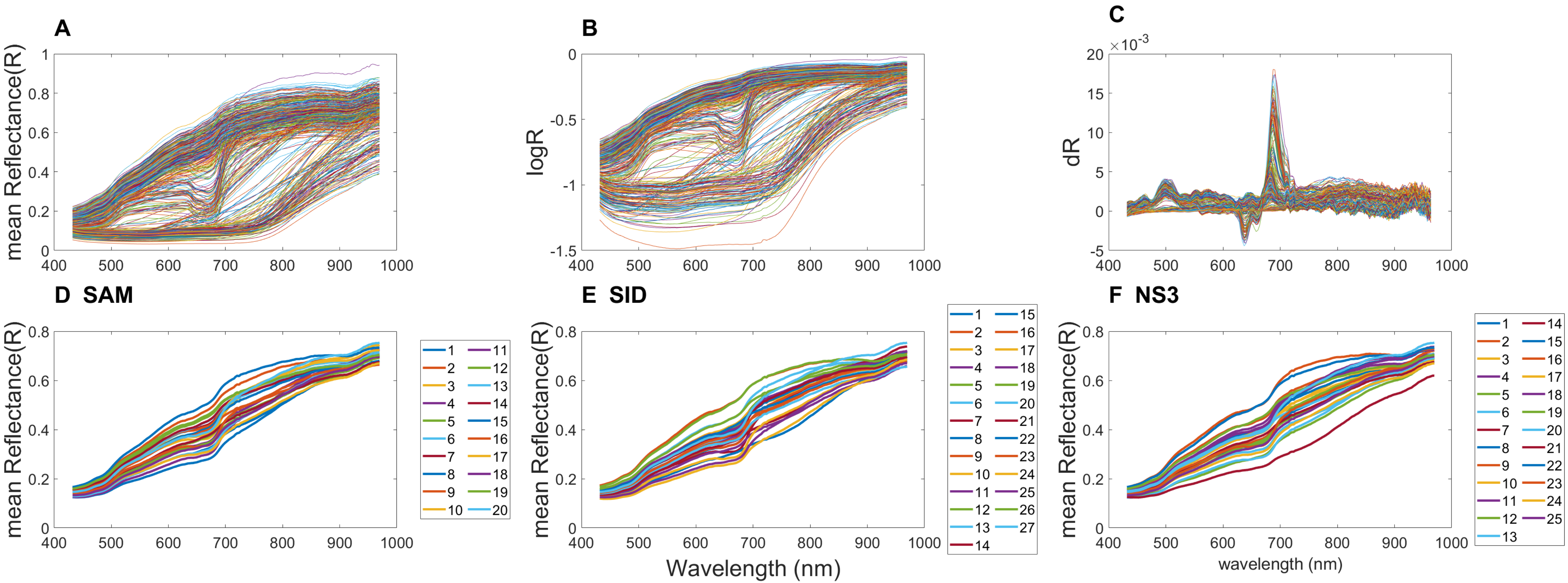

Supplementary Figure S3. Averaged reflectances (A), and their logarithmic transformations (B) and first derivatives (C) of KSCC accession. In panels D, E, and F, spectral similarity was calculated using SAM (D), SID (E), and NS3(F) of averaged reflectance from A and categorized into different groups using hierarchical clustering.

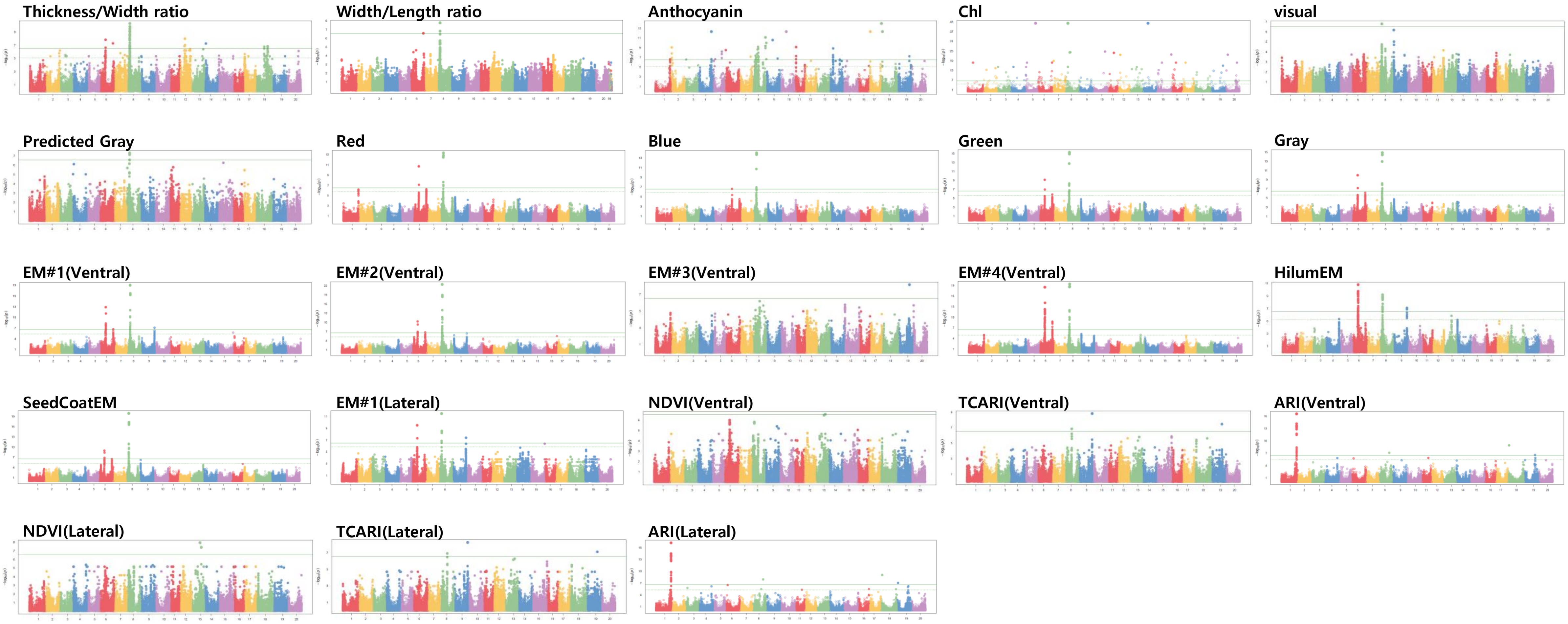

Supplementary Figure S4. Manhattan plots of the 23 features used in the machine learning LDA analysis.
